# Cembrane Derivatives from the Soft Corals, *Sinularia gaweli* and *Sinularia flexibilis*

**DOI:** 10.3390/md11062154

**Published:** 2013-06-17

**Authors:** Li-Chung Hu, Wei-Hsuan Yen, Jui-Hsin Su, Michael Yen-Nan Chiang, Zhi-Hong Wen, Wu-Fu Chen, Ting-Jang Lu, Yu-Wei Chang, Yung-Husan Chen, Wei-Hsien Wang, Yang-Chang Wu, Ping-Jyun Sung

**Affiliations:** 1 Graduate Institute of Marine Biotechnology and Department of Life Science and Institute of Biotechnology, National Dong Hwa University, Pingtung 944, Taiwan; E-Mails: stoja582@gmail.com (L.-C.H.); xyz78714@hotmail.com (W.-H.Y.); x2219@nmmba.gov.tw (J.-H.S.); 2 National Museum of Marine Biology and Aquarium, Pingtung 944, Taiwan; E-Mails: tony_chen72001@yahoo.com.tw (Y.-H.C.); whw@nmmba.gov.tw (W.-H.W.); 3 Department of Chemistry, National Sun Yat-sen University, Kaohsiung 804, Taiwan; E-Mail: michael@mail.nsysu.edu.tw; 4 Department of Marine Biotechnology and Resources and Asia-Pacific Ocean Research Center, National Sun Yat-sen University, Kaohsiung 804, Taiwan; E-Mail: wzh@mail.nsysu.edu.tw; 5 Department of Neurosurgery, Kaohsiung Chang Gung Memorial Hospital and Chang Gung University College of Medicine, Kaohsiung 833, Taiwan; E-Mail: ma4949@adm.cgmh.org.tw; 6 Graduate Institute of Food Science and Technology, National Taiwan University, Taipei 106, Taiwan; E-Mail: tjlu@ntu.edu.tw; 7 Department of Food Science, National Taiwan Ocean University, Keelung 202, Taiwan; E-Mail: bweichang@mail.ntou.edu.tw; 8 School of Pharmacy, College of Pharmacy, China Medical University, Taichung 404, Taiwan; 9 Chinese Medicine Research and Development Center, China Medical University Hospital, Taichung 404, Taiwan; 10 Center for Molecular Medicine, China Medical University Hospital, Taichung 404, Taiwan; 11 Graduate Institute of Natural Products, Kaohsiung Medical University, Kaohsiung 807, Taiwan

**Keywords:** cembrane, norcembrane, *Sinularia flexibilis*, *Sinularia gaweli*, X-ray, anti-inflammatory activity

## Abstract

A new norcembranoidal diterpene, 1-*epi*-sinulanorcembranolide A (**1**), and a new cembranoidal diterpene, flexibilin D (**2**), were isolated from the soft corals, *Sinularia gaweli* and *Sinularia flexibilis*, respectively. The structures of new metabolites **1** and **2** were elucidated by spectroscopic methods, and compound **2** was found to significantly inhibit the accumulation of the pro-inflammatory iNOS and COX-2 proteins of the lipopolysaccharide (LPS)-stimulated RAW264.7 macrophage cells. In addition, *S. flexibilis* yielded a known cembrane, 5-dehydrosinulariolide (**3**); the structure, including its absolute stereochemistry, was further confirmed by single-crystal X-ray diffraction analysis.

## 1. Introduction

**Figure 1:**
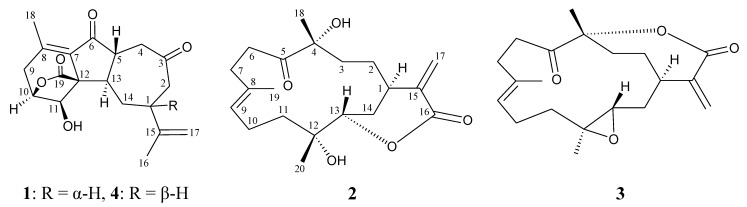
The structures of 1-*epi*-sinulanorcembranolide A (**1**), flexibilin D (**2**), 5-dehydrosinulariolide (**3**) and sinulanorcembranolide A (**4**).

Octocorals belonging to the genus *Sinularia*, which are distributed widely in the tropical and subtropical Indo-Pacific Ocean, have been proven to be rich sources of bioactive terpenoid analogues [1,2,3,4]. In ongoing studies on the chemical constituents from marine invertebrates collected off the waters of Taiwan, the intersection of the Kuroshio current and the South China Sea surface current, the octocorals *Sinularia gaweli* and *Sinularia flexibilis* (phylum Cnidaria, class Anthozoa, subclass Octocorallia, order Alcyonacea, family Alcyoniidae) were studied, as their organic extract was found to show meaning signals in NMR studies. In previous studies, a series of interesting secondary metabolites were isolated from the octocorals, *S. gaweli* [5,6,7] and *S. flexibilis* [8,9,10,11,12,13,14,15,16,17,18], collected in the waters of Taiwan. We further isolated a new norcembranoidal diterpene, 1-*epi*-sinulanorcembranolide A (**1**), from *S. gaweli* and a new cembranoidal diterpene, flexibilin D (**2**), along with a known cembrane, 5-dehydrosinulariolide (**3**) [10,13,19,20,21,22,23], from *S. flexibilis* (Figure 1). In this paper, we describe the isolation, structure determination and bioactivity of terpenoids **1**–**3**. 

## 2. Results and Discussion

### 2.1. Isolation and Structure Determination of 1-*epi*-Sinulanorcembranolide A (1) from *Sinularia gaweli*

1-*epi*-Sinulanorcembranolide A (**1**) was isolated as an amorphous powder that gave a pseudomolecular ion [M + Na]^+^ at *m/z* 353.1368 in the HRESIMS, indicating the molecular formula, C_19_H_22_O_5_ (calcd. for C_19_H_22_O_5_Na, 353.1365), and implying nine degrees of unsaturation. The IR spectrum of **1** showed strong bands at 3448, 1769, 1701 and 1687 cm^–1^, consistent with the presence of hydroxy, ester, ketone and α,β-unsaturated ketone groups. The ^13^C NMR and DEPT spectra of **1** showed that this compound had 19 carbons (Table 1), including two methyls, four sp^3^ methylenes, five sp^3^ methines, an sp^3^ quaternary carbon, an sp^2^ methylene and six sp^2^ quaternary carbons. From the ^13^C NMR spectrum (Table 1), **1** was found to possess two keto carbonyls (*δ*_C_ 208.7, C-3; 198.0, C-6) and an ester group (*δ*_C_ 174.7, C-19). Two additional unsaturated functionalities were indicated by ^13^C resonances at *δ*_C_ 147.1 (C-15), 145.6 (C-8), 129.9 (C-7) and 112.3 (CH_2_-17), suggesting the presence of a tetrasubstituted olefin and an exocyclic carbon-carbon double bond. Thus, from the above data, five degrees of unsaturation were accounted for, and **1** was identified as a tetracyclic compound.

**Table 1:** ^1^H (500 MHz, CDCl_3_) and ^13^C (125 MHz, CDCl_3_) NMR data and ^1^H–^1^H COSY and HMBC correlations for norcembrane **1**.

Position	*δ*_H_ (*J* in Hz)	*δ*_C_, Multiple	^1^H–^1^H COSY	HMBC
1	2.79 m	39.4, CH	H_2_-2, H_2_-14	C-3, -13, -14, -15
2/2′	2.71 d (16.0); 2.47 dd (16.0, 7.0)	42.8, CH_2_	H-1	C-1, -3, -4, -14, -15
3		208.7, C		
4/4′	3.00 dd (15.5, 11.0); 2.56 (15.5, 7.0)	38.9, CH_2_	H-5	C-3, -5, -6, -13
5	2.82 m	46.6, CH	H_2_-4, H-13	C-3, -4, -12, -13, -14
6		198.0, C		
7		129.9, C		
8		145.6, C		
9	2.68 br s	39.1, CH_2_	H-10	C-7, -8
10	4.50 dd (2.5, 2.5)	79.4, CH	H_2_-9, H-11	C-8, -11, -19
11	4.15 s	77.0, CH	H-10	C-7, -10, -19
12		52.6, C		
13	2.88 m	32.9, CH	H-5, H_2_-14	C-1, -5, -19
14/14′	2.71 br d (5.5); 2.11 m	28.4, CH_2_	H-1, H-13	C-1, -5, -12, -15
15		147.1, C		
16	1.76 s	21.5, CH_3_	H-17a	C-1, -15, -17
17a/b	4.86 s; 4.71 s	112.3, CH_2_	H_3_-16	C-1, -16
18	2.02 s	21.0, CH_3_		C-7, -8, -9
19		174.7, C		

**Figure 2:**
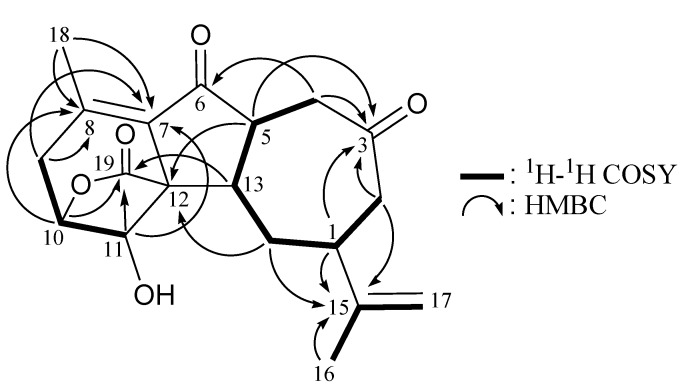
^1^H–^1^H COSY and selected HMBC correlations (protons→quaternary carbons) for **1**.

From the ^1^H–^1^H COSY spectrum of **1 ** (Table 1 and Figure 2), it was possible to differentiate among the separate spin systems of H_2_-4/H-5/H-13/H_2_-14/H-1/H_2_-2, H_2_-9/H-10/H-11 and H_3_-16/H-17a (by allylic coupling). These data, together with the key HMBC correlations between protons and quaternary carbons (Table 1 and Figure 2), such as H-1, H_2_-2, H-4, H-5/C-3; H_2_-4/C-6; H_2_-9, H-11, H_3_-18/C-7; H_2_-9, H-10, H_3_-18/C-8; H-5, H-14′/C-12; H-1, H_2_-2, H_2_-14, H_3_-16/C-15; and H-10, H-11, H-13/C-19, established the main carbon skeleton of **1**. The vinyl methyls at C-8 and C-15 were confirmed by the HMBC correlations between H_3_-16/C-1, -15, -17 and H_3_-18/C-7, -8, -9. Thus, based on the above findings, **1** was revealed as a norcembranoidal diterpene possessing a γ-lactone moiety.

**Figure 3:**
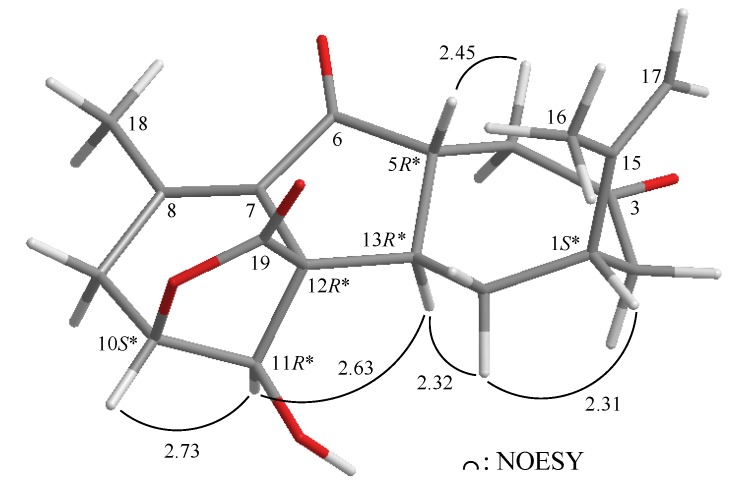
The computer-generated model of **1** using molecular mechanics calculations (MM2) force field calculations and the calculated distances (Å) between selected protons with key NOESY correlations.

The relative configuration of **1** was elucidated mainly from a NOESY spectrum as being compatible with that of **1**, ascertained using molecular mechanics calculations (MM2) [24]. In the NOESY experiment of **1** (Figure 3), one of the C-14 methylene protons (*δ*_H_ 2.11) exhibited correlations with H-1 and H-13, but not with H-5, indicated that these protons were situated on the same face and were assigned as α protons, since H-5 is a β-substituent at C-5. H-11 showed correlations with H-10 and H-13, as well as the lack of coupling detected between H-10 and H-11, indicating that the dihedral angle between H-10 and H-11 is approximately 90° and that the lactone moiety and 11-hydroxy group were β-oriented by molecular modeling analysis. From the above evidence, the relative configurations of the chiral carbons of **1** were assumed to be 1S*, 5R*, 10S*, 11R*, 12R* and 13R*. By comparison of the spectral data with those of a known norcembranoidal diterpene, sinulanorcembranolide A (**4**) (Figure 1), which was also isolated from *S. gaweli* [7], **1** was found to be the 1-*epi*-compound of **4** and should be named as 1-*epi*-sinulanorcembranolide A. To the best of our knowledge, compounds **1** and **4** are the only two norcembranoidal diterpenes with carbon-carbon linkages between C-5/13 and C-7/12. A plausible biosynthetic pathway for the compounds of this type was proposed in our previous study [7].

### 2.2. Isolation and Structure Determination of Flexibilin D (2) from *Sinularia flexibilis*

Flexibilin D (**2**) was obtained as a white powder. The molecular formula of **2** was established as C_20_H_30_O_5_ (6° of unsaturation) from a sodiated molecule at *m/z* 373 in the ESIMS spectrum and further supported by HRESIMS (*m/z* 373.1988, calcd. for C_20_H_30_O_5_Na, 373.1991). The IR spectrum of **2** exhibited the presence of hydroxy (ν_max_ 3422 cm^−1^) and carbonyl (ν_max_ 1712 cm^−1^) groups. From the ^13^C NMR data of **2** (Table 2), a suite of resonances at *δ*_C_ 165.5 (C-16), 138.0 (C-15), 128.4 (CH_2_-17), 79.1 (CH-13), 35.4 (CH-1) and 26.2 (CH_2_-14) could be assigned to the α-exomethylene-δ-lactone moiety. Two additional unsaturated functionalities were indicated by ^13^C resonances at *δ*_C_ 213.8 (C-5), 134.8 (C-8) and 126.1 (CH-9), suggesting the presence of a keto carbonyl and a trisubstituted olefin. On the basis of the overall unsaturation data, **2** was concluded to be a diterpenoid molecule possessing two rings. The ^1^H NMR spectrum of **2** showed the presence of three methyl groups: two singlets at *δ*_H_ 1.35 and 1.31, representing the methyl groups on oxygenated quaternary carbons, and a vinyl methyl at *δ*_H_ 1.66.

**Table 2:** ^1^H (400 MHz, CDCl_3_) and ^13^C (100 MHz, CDCl_3_) NMR data and ^1^H–^1^H COSY and HMBC correlations for cembrane **2**.

Position	*δ*_H_ (*J* in Hz)	*δ*_C_, Multiple	^1^H–^1^H COSY	HMBC
1	2.85 m	35.4, CH	H_2_-2, H_2_-14	n.o. *^a^*
2/2′	1.49 m; 1.32 m	28.2, CH_2_	H-1, H_2_-3	C-1, -3, -4, -14, -15
3/3′	1.79 ddd (13.6, 11,2, 5.6); 1.64 m	37.2, CH_2_	H_2_-2	C-1, -2, -4, -5, -18
4		78.9, C		
5		213.8, C		
6/6′	2.76 ddd (18.0, 8.0, 3.2); 2.65 ddd (18.0, 10.2, 3.2)	35.1, CH_2_	H_2_-7	C-5, -7, -8
7/7′	2.48 m; 2.33 m	31.8, CH_2_	H_2_-6	C-5, -6, -8, -9
8		134.8, C		
9	5.15 dd (5.6, 5.6)	126.1, CH	H_2_-10, H_3_-19	C-7, -10, -19
10	2.18 m	23.1, CH_2_	H-9, H_2_-11	C-8, -9, -11, -12
11/11′	1.87 dd (8.4, 2,8); 1.69 m	36.7, CH_2_	H_2_-10	C-9, -12, -13
12		73.9, C		
13	4.29 dd (9.2, 6.0)	79.1, CH	H_2_-14	C-1, -14, -20
14	1.91 m	26.2, CH_2_	H-1, H-13	C-12, -13, -15
15		138.0, C		
16		165.5, C		
17a/b	6.43 d (1.2); 5.56 dd (1.2, 1.2)	128.4, CH_2_		C-1, -15, -16
18	1.35 s	25.2, CH_3_		C-3, -4, -5
19	1.66 s	17.2, CH_3_	H-9	C-7, -8, -9
20	1.31 s	24.1, CH_3_		C-11, -12, -13
OH-4	3.24 br s			C-5

*^a^* n.o. = not observed.

**Figure 4:**
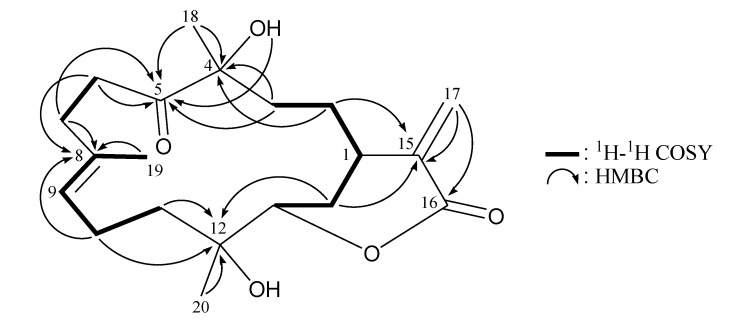
^1^H–^1^H COSY and selected HMBC correlations (protons→quaternary carbons) for cembrane **2**.

The ^1^H NMR coupling information in the ^1^H–^1^H COSY spectrum of **2** enabled identification of the H-13/H_2_-14/H-1/H_2_-2/H_2_-3, H_2_-6/H_2_-7, H-9/H_2_-10/H_2_-11 and H-9/H_3_-19 (by allylic coupling) units, which were assembled with the assistance of an HMBC experiment (Table 2 and Figure 4), enabling establishment of the main carbon skeleton of **2**. A vinyl methyl at C-8 was confirmed by the allylic coupling between H-9/H_3_-19 in the ^1^H–^1^H COSY spectrum and by the HMBC correlations between H_3_-19/C-7, -8, -9; and H-9/C-19. The tertiary methyls at C-4 and C-12 were confirmed by the HMBC correlations between H_3_-18/C-3, -4, -5 and H_3_-20/C-11, -12, -13.

**Figure 5:**
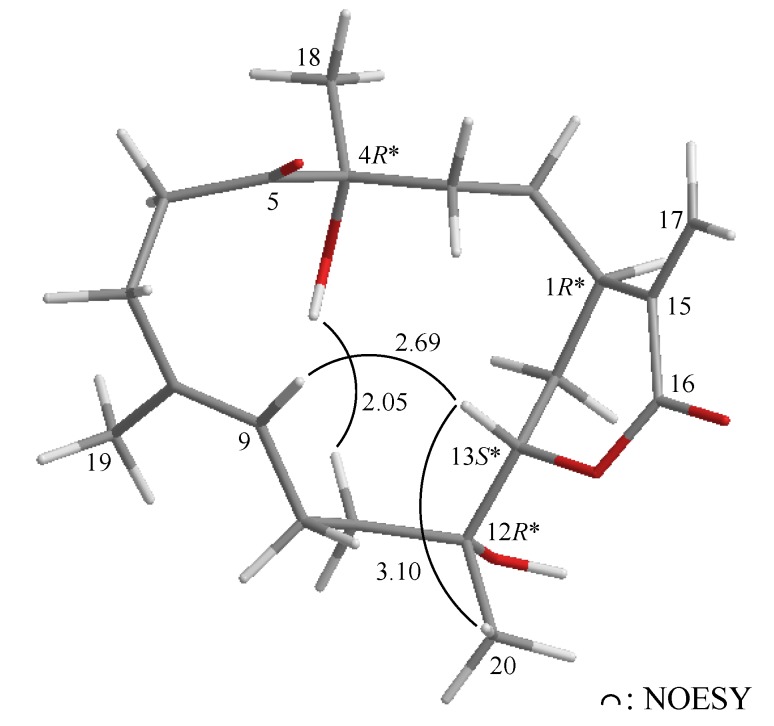
The computer-generated model of **2** using MM2 force field calculations and the calculated distances (Å) between selected protons with key NOESY correlations.

The relative stereochemistry of **2** was elucidated by the analysis of NOE correlations, as shown in Figure 5. In the NOESY experiment of **2**, H-13 was found to be correlated with H_3_-20, but not with H-1; this demonstrated that H-1, H-13 and Me-20 were α-, β- and β-oriented, respectively. Additionally, correlations between H-9 and H-13, and the absence of correlation between H-9/H_3_-19, reflected the *E* geometry of the double bond at C-8/9. The 4-hydroxy proton was found to be correlated with one of the C-11 methylene protons (*δ*_H_ 1.69), indicating that the 4-hydroxy group was α-oriented. Based on the above findings, the structure of **2** was elucidated, and the chiral carbons of **2** were assigned as 1R*, 4R*, 12R* and 13S*. 

**Figure 6:**
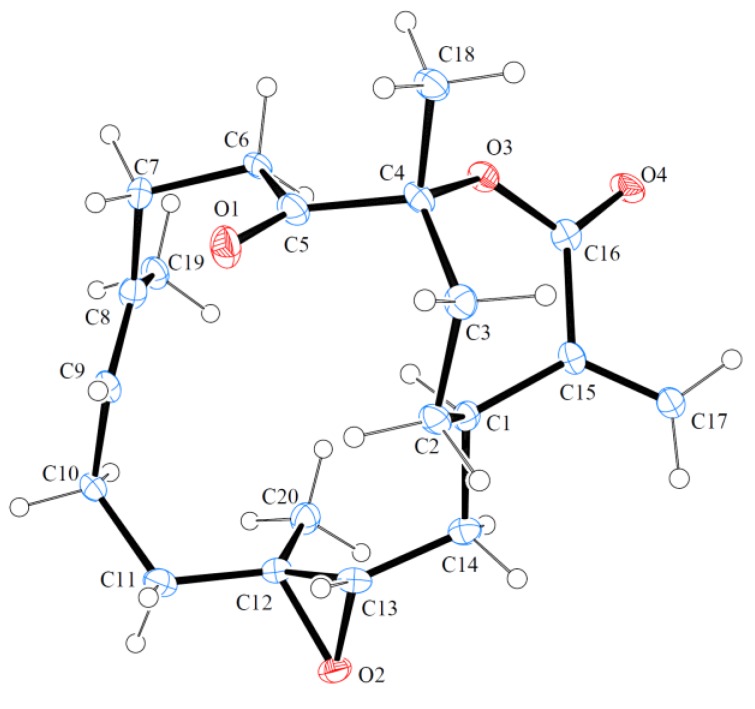
Molecular plot of **3** with confirmed absolute configuration.

The known cembrane, 5-dehydrosinulariolide (=11-dehydrosinulariolide) (**3**) [10,13,19,20,21,22,23], was first isolated from an Indonesian soft coral, *Sinularia flexibilis* [19], and its structure was elucidated by spectroscopic and chemical methods and confirmed by single-crystal X-ray diffraction analysis [13,20]. In this study, the absolute stereochemistry of **3** was established by single-crystal X-ray diffraction analysis for the first time (Figure 6). The configurations for all chiral carbons of **3** were assigned as 1*R*, 4*R*, 12*S* and 13*S*. The proton and carbon chemical shifts for **3** were reassigned by detailed analyses of 1D and 2D NMR spectra (Table 3).

**Table 3:** ^1^H (400 MHz, CDCl_3_) and ^13^C (100 MHz, CDCl_3_) NMR data and ^1^H–^1^H COSY and HMBC correlations for cembrane **3**.

Position	*δ*_H_ (*J* in Hz)	*δ*_C_, Multiple	^1^H–^1^H COSY	HMBC
1	1.81 m	34.7, CH	H_2_-2, H_2_-14	C-14, -15, -16, -17
2/2′	2.24 ddd (18.0, 12.4, 6.0); 1.17 m	30.8, CH_2_	H-1, H_2_-3	C-1, -3, -4, -14, -15
3/3′	2.41 dd (15.6, 6.0); 1.87 m	33.1, CH_2_	H_2_-2	C-1, -2, -4, -18
4		90.4, C		
5		209.1, C		
6/6′	3.11 ddd (20.4, 10.8, 1.6); 2.63 ddd (20.4, 8.4, 1.6)	33.5, CH_2_	H_2_-7	C-5, -7, -8
7/7′	2.68 m; 1.94 m	29.9, CH_2_	H_2_-6	C-5, -6, -8, -9
8		134.9, C		
9	5.02 ddq (7.2, 7.2, 1.2)	122.6, CH	H_2_-10, H_3_-19	C-7, -10, -19
10	2.13 m	24.3, CH_2_	H-9, H_2_-11	C-8, -9, -11, -12
11/11′	2.02 ddd (13.6, 4.4, 4.0); 1.11 m	37.4, CH_2_	H_2_-10	C-9, -10, -12, -13
12		60.5, C		
13	2.65 br s	62.1, CH	H_2_-14	C-14
14/14′	1.84 br s; 1.44 dd (22.8, 10.8)	32.4, CH_2_	H-1, H-13	C-1, -2, -12, -13, -15
15		143.5, C		
16		167.5, C		
17a/b	6.26 s; 5.45 s	125.7, CH_2_		C-1, -15, -16
18	1.43 s	29.5, CH_3_		C-3, -4, -5
19	1.59 d (1.2)	17.1, CH_3_	H-9	C-7, -8, -9
20	1.13 s	16.0, CH_3_		C-11, -12, -13

**Figure 7:**
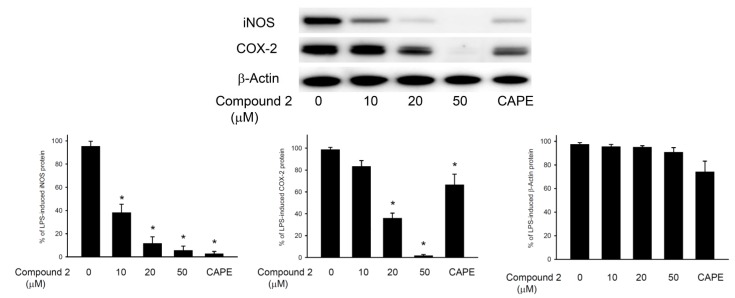
Effects of compound **2** on iNOS and COX-2 protein expression of RAW264.7 macrophage cells by immunoblot analysis. The values are the mean ± SEM (*n* = 5). Relative intensity of the lipopolysaccharide (LPS)-alone stimulated group was taken as 100%. Under the same experimental condition, CAPE (caffeic acid phenylethyl ester, 10 μM), reduces the levels of the iNOS and COX-2 to 2.8 ± 4.6 and 66.7% ± 9.6%, respectively. * Significantly different from LPS-alone stimulated group (*p* < 0.05).

The *in vitro* anti-inflammatory effects of compounds **1**–**3** were tested. In this assay, the upregulation of the pro-inflammatory iNOS (inducible nitric oxide synthase) and COX-2 (cyclooxygenase-2) proteins expression of LPS (lipopolysaccharide)-stimulated RAW264.7 macrophage cells was evaluated using immunoblot analysis. At a concentration of 20 μM, compound **2** was found to significantly reduce the levels of iNOS and COX-2 to 19.27 ± 2.72 and 30.08% ± 9.07%, respectively, relative to the control cells stimulated with LPS only (Figure 7). Levels of β-actin protein (internal control) demonstrated no significant difference among concentrations of 10, 20 and 50 μM of compound **2** or compared with LPS only. Thus, compound **2** might be promising as an anti-inflammatory agent, as it does not exhibit cytotoxicity to RAW264.7 macrophage cells. Metabolites **1** and **3** did not attenuate the iNOS and COX-2 expression in LPS-stimulated macrophage cells at concentrations of 10 and 20 μM.

## 3. Experimental Section

### 3.1. General Experimental Procedures

Optical rotations were measured on a Jasco P-1010 digital polarimeter (Japan Spectroscopic Corporation, Tokyo, Japan). Infrared spectra were recorded on a Varian Diglab FTS 1000 FT-IR spectrometer (Varian Inc., Palo Alto, CA, USA); peaks are reported in cm^–1^. NMR spectra were recorded on a Varian Inova 500 spectrometer (Varian Inc., Palo Alto, CA, USA)or a Varian Mercury Plus 400 NMR spectrometer (Varian Inc., Palo Alto, CA, USA) using the residual CHCl_3_ signal (*δ*_H_ 7.26 ppm) as the internal standard for ^1^H NMR and CDCl_3_ (*δ*_C_ 77.1 ppm) for ^13^C NMR. Coupling constants (*J*) are given in Hz. ESIMS and HRESIMS were recorded using a Bruker APEX II mass spectrometer (Bruker, Bremen, Germany). Column chromatography was performed on silica gel (230–400 mesh, Merck, Darmstadt, Germany). TLC was carried out on precoated Kieselgel 60 F_254_ (0.25 mm, Merck, Darmstadt, Germany); spots were visualized by spraying with 10% H_2_SO_4_ solution, followed by heating. The normal phase HPLC (NP-HPLC) was performed using a system comprised of a Hitachi L-7100 pump, a Hitachi L-7455 photodiode array detector (Hitachi Ltd. Tokyo, Japan), a Rheodyne 7725 injection port (Rheodyne LLC., Rohnert Park, CA, USA) and a normal phase column (Hibar 250 × 10 mm, silica gel 60, 5 μm, Merck, Darmstadt, Germany). The reverse phase HPLC (RP-HPLC) was performed using a system comprised of a Hitachi L-7100 pump, a Hitachi L-2455 photodiode array detector (Hitachi Ltd. Tokyo, Japan), a Rheodyne 7725 injection port (Rheodyne LLC. Rohnert Park, CA, USA) and a reverse phase column (Varian Polaris C18-A, 250 × 10 mm, 5 μm, Varian Inc., Palo Alto, CA, USA).

### 3.2. Animal Material

#### 3.2.1. *Sinularia gaweli*

Specimens of the octocoral, *S. gaweli*, were collected by hand using scuba equipment off the coast of Sansiantai, Taitung County, Taiwan in October, 2011, and stored in a freezer (−20 °C) until extraction. A voucher specimen (NMMBA-TWSC-11007) was deposited in the National Museum of Marine Biology and Aquarium, Taiwan.

#### 3.2.2. *Sinularia flexibilis*

Specimens of the octocoral, *S. flexibilis*, were collected by hand using scuba equipment off the coast of Southern Taiwan in July, 2011, and stored in a freezer (−20 °C) until extraction. A voucher specimen (NMMBA-TWSC-11005) was deposited in the National Museum of Marine Biology and Aquarium, Taiwan.

### 3.3. Extraction and Isolation

#### 3.3.1. *Sinularia gaweli*

Specimens of the soft coral, *S. gaweli* (wet weight 1.30 kg, dry weight 328 g), were extracted with ethyl acetate (EtOAc). The EtOAc extract left after removal of the solvent (11.4 g) was separated by silica gel and eluted using *n*-hexane/EtOAc in a stepwise fashion from 100:1–pure EtOAc to yield 13 fractions, A–M. Fraction I was separated by NP-HPLC, using a mixture of *n*-hexane and acetone (5:1) to yield 8 subfractions, 1–8. Fraction I6 was purified by RP-HPLC, using a mixture of methanol and H_2_O (1:1, flow rate: 1.0 mL/min) as the mobile phase to afford **1** (2.0 mg, *t*_R_ = 33 m).

1-*epi*-Sinulanorcembranolide A (**1**): white powder; mp 120–122 °C; [α]^25^_D_ −46 (*c* 0.1, CHCl_3_); IR (neat) ν_max_ 3448, 1769, 1701, 1687 cm^–1^; ^1^H (500 MHz, CDCl_3_) and ^13^C (125 MHz, CDCl_3_) NMR data, see Table 2; ESIMS: *m/z* 353 [M + Na]^+^; HRESIMS: *m/z* 353.1368 (calcd. for C_19_H_22_O_5_Na, 353.1365).

#### 3.3.2. *Sinularia flexibilis*

Sliced bodies of the soft coral, *S. flexibilis* (wet weight 3.0 kg, dry weight 950 g), were extracted with ethyl acetate (EtOAc). The EtOAc extract was separated by silica gel and eluted using *n*-hexane/EtOAc in a stepwise fashion from pure *n*-hexane–100:1–pure EtOAc to yield 11 fractions, A–K. Fraction H was repurified by NP-HPLC using a mixture of *n*-hexane and acetone (7:1, flow rate: 2.0 mL/min) as the mobile phase to afford **3** (58.7 mg, *t*_R_ = 240 m). Fraction J was chromatographed on silica gel and eluted using *n*-hexane/EtOAc (stepwise, 2:1–1:1) to afford subfractions 1–10. Fraction J7 was separated by NP-HPLC using a mixture of *n*-hexane and acetone (2:1, flow rate: 2.0 mL/min) as the mobile phase to afford **2** (3.1 mg, *t*_R_ = 85 m). 

Flexibilin D (**2**): white powder; mp 107–109 °C; [α]^25^_D_ −16 (*c* 0.2, CHCl_3_); IR (neat) ν_max_ 3422, 1712 cm^–1^; ^1^H (400 MHz, CDCl_3_) and ^13^C (100 MHz, CDCl_3_) NMR data, see Table 2; ESIMS: *m/z* 373 [M + Na]^+^; HRESIMS: *m/z* 373.1988 (calcd. for C_20_H_30_O_5_Na, 373.1991).

5-Dehydrosinulariolide (**3**): white powder; mp 109–111 °C ( 117–119 °C [10]; 120 °C [20]; 112–115 °C [21]); [α]^25^_D_ +93 (*c* 0.79, CHCl_3_) ( [α]^24^_D_ +95.5 (*c* 0.16, CHCl_3_) [10]; [α]_D_ +87 (EtOH) [20]; [α]^25^_D_ +45.1 (*c* 5.5, CHCl_3_) [21]); IR (neat) ν_max_ 3411, 1713 cm^–1^; ^1^H (400 MHz, CDCl_3_) and ^13^C (100 MHz, CDCl_3_) NMR data, see Table 3; ESIMS: *m/z* 355 [M + Na]^+^. 

### 3.4. Single-Crystal X-ray Crystallography of 5-Dehydrosinulariolide (**3**) [25]

Suitable colorless prisms of **3** were obtained from a solution of ethyl acetate. Crystal data and experimental details: C_20_H_28_O_4_, *M_r_* = 332.42, crystal size 0.12 × 0.10 × 0.05 mm, crystal system monoclinic, space group *P*2_1_ (#4), with *a* = 9.3510(5) Å, *b* = 10.8986(5) Å, *c* = 17.6895(8) Å, β = 101.873(4)°, *V* = 1764.22(15) Å^3^, , *Z* = 4, *D*_calcd_ = 1.252 g/cm^3^, λ (Cu, kα) = 1.54178 Å. Intensity data were measured on a Bruker APEX-II CCD diffractometer equipped with a micro-focus Cu radiation source and a Montel mirror up to *θ*_max_ of 26° at 100 K. All 4234 reflections were collected. The structure was solved by direct methods and refined by a full-matrix least-squares procedure. The refined structural model converged to a final *R*1 = 0.0810, *wR*2 = 0.2220 for 4023 observed reflection [*I* > 2σ(*I*)] and 433 variable parameters. The absolute configuration was determined by Flack’s method with Flack’s parameter determined as 0.00(45) [26].

### 3.5. Molecular Mechanics Calculations

Implementation of the MM2 force field [24] in CHEM3D PRO software from CambridgeSoft Corporation (Cambridge, MA, USA; ver. 9.0, 2005) was used to calculate molecular models.

### 3.6. *In Vitro* Anti-Inflammatory Assay

The macrophage (RAW264.7) cell line was purchased from ATCC. *In vitro* anti-inflammatory activity of compounds **1** and **2** was measured by examining the inhibition of lipopolysaccharide (LPS)-induced upregulation of iNOS (inducible nitric oxide synthase) and COX-2 (cyclooxygenase-2) proteins in macrophage cells using Western blotting analysis [27,28,29].

## 4. Conclusions

In the present study, a new norcembranoidal diterpene, 1-*epi*-sinulanorcembranolide A (**1**), and a new cembranoidal diterpene, flexibilin D (**2**), were isolated from the soft corals, *Sinularia gaweli* and *Sinularia flexibilis*, respectively. In addition, a known cembrane, 5-dehydrosinulariolide (=11-dehydrosinulariolide) (**3**), which was found to show antitumor activities toward CAL-27 (human oral adenosquamous carcinoma) [30] and A2058 (human Caucasian metastatic melanoma) [31] cells and displayed neuroprotection in an *in vitro* Parkinson’s model [32], was also obtained from *S. flexibilis.* The structure, including its absolute stereochemistry of **3** was further confirmed by single-crystal X-ray diffraction analysis for the first time in this study. Moreover, in the anti-inflammatory test, compound **2** was found to significantly inhibit the accumulation of the pro-inflammatory iNOS and COX-2 proteins of the LPS-stimulated RAW264.7 macrophage cells. Because octocorals are claimed to be endangered species and based on the potential medicinal use, the soft coral *S. flexibilis* has begun to be transplanted to culturing tanks located in the National Museum of Marine Biology and Aquarium, Taiwan, for exhibition and the extraction of additional natural products to establish a stable supply of bioactive material. 
